# XPO1 in B cell hematological malignancies: from recurrent somatic mutations to targeted therapy

**DOI:** 10.1186/s13045-017-0412-4

**Published:** 2017-02-14

**Authors:** Vincent Camus, Hadjer Miloudi, Antoine Taly, Brigitte Sola, Fabrice Jardin

**Affiliations:** 1 Normandie Univ, INSERM U1245, UNICAEN, UNIROUEN, Caen, France; 2 Department of Hematology, Centre Henri Becquerel, Rouen, France; 3 Laboratoire de Biochimie Théorique, Institut de Biologie Physico-Chimique, CNRS, Université Paris Diderot, Paris, France

**Keywords:** XPO1, Exportin, Lymphoma, Targeted therapy, Minimal residual disease

## Abstract

Many recent publications highlight the large role of the pivotal eukaryotic nuclear export protein exportin-1 (XPO1) in the oncogenesis of several malignancies, and there is emerging evidence that XPO1 inhibition is a key target against cancer. The clinical validation of the pharmacological inhibition of XPO1 was recently achieved with the development of the selective inhibitor of nuclear export compounds, displaying an interesting anti-tumor activity in patients with massive pre-treated hematological malignancies. Recent reports have shown molecular alterations in the gene encoding XPO1 and showed a mutation hotspot (E571K) in the following two hematological malignancies with similar phenotypes and natural histories: primary mediastinal diffuse large B cell lymphoma and classical Hodgkin’s lymphoma. Emerging evidence suggests that the mutant *XPO1* E571K plays a role in carcinogenesis, and this variant is quantifiable in tumor and plasma cell-free DNA of patients using highly sensitive molecular biology techniques, such as digital PCR and next-generation sequencing. Therefore, it was proposed that the *XPO1* E571K variant may serve as a minimal residual disease tool in this setting. To clarify and summarize the recent findings on the role of XPO1 in B cell hematological malignancies, we conducted a literature search to present the major publications establishing the landscape of *XPO1* molecular alterations, their impact on the XPO1 protein, their interest as biomarkers, and investigations into the development of new XPO1-targeted therapies in B cell hematological malignancies.

## Background

Exportin-1, also called chromosome region maintenance 1 (CRM1/XPO1), is a pivotal eukaryotic nuclear export protein that carries an extensive array of proteins from the nucleus to the cytoplasm. XPO1 is a member of the importin-β superfamily of karyopherins that mediates the translocation of numerous RNAs and cellular regulatory proteins, including tumor suppressor proteins (TSPs), such as p53, BRCA1, survivin, nucleophosmin, APC, and FOXO. XPO1 hydrophobic groove binds to the leucine-rich nuclear export signal (NES) domain of these “cargo” proteins. XPO1 is overactive in many cancer [1–7], and thus, XPO1 has been considered a potential anti-cancer target for decades.

The clinical validation of the pharmacological inhibition of XPO1 was recently achieved with the development of the selective inhibitor of nuclear export (SINE) compounds. SINEs are orally bioavailable small-molecule inhibitors of XPO1-mediated nuclear protein export [8–10].

Especially for hematological malignancies, many publications have highlighted the value and efficacy of several SINE agents, all of which have an interesting anti-tumor activity in patients with massive pre-treated hematological malignancies [2, 8, 9, 11–15]. Currently, the dominant SINE pharmacological agent, selinexor (KPT-330), is being assessed in phase 1 and 2 clinical trials for various cancers.

Furthermore, recent studies have focused on the characterization of the molecular alterations in the gene encoding XPO1 and showed a mutation hotspot (E571K) in the following two hematological malignancies with similar phenotypes and natural histories: primary mediastinal diffuse large B cell lymphoma (PMBL) and classical Hodgkin’s lymphoma (cHL) [12, 16]. Missense substitutions targeting *XPO1* have also been previously reported at a low frequency (<5%) in chronic lymphocytic leukemia (CLL) and esophageal squamous cell carcinoma (ESCC), indicating that these mutations may also play a role in other oncogenic processes [17–19].

We conducted a literature search to present the recent major publications establishing the landscape of *XPO1* molecular alterations, their impact on the XPO1 protein, their interest as biomarkers, and investigations into the development of new SINE therapies in B cell hematological malignancies.

## *XPO1* gene and XPO1 protein

The XPO1 protein was first identified by a genetic screening of *Saccharomyces pombe* and was recognized as a nuclear component maintaining the higher order of the chromosome structure [20]. Thereafter, XPO1 was described as a ubiquitous nuclear export protein of the karyopherin β family [21–23]. The human *XPO1* gene is located on chromosome 2p15, which is close to the c-*REL* 2p16.1 locus, a locus well known to be gained or amplified in PMBL, germinal B-cell-like (GCB) diffuse large B cell lymphoma (DLBCL), and cHL.

**Fig. 1:**
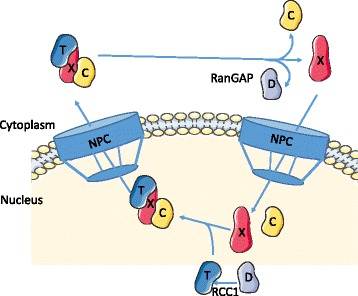
Schematic representation of XPO1-mediated nuclear export. RanGDP (*D*) is converted to RanGTP (*T*) through the action of RCC1 (the Ran guanine exchange factor). The export complex composed of RanGTP, cargo (*C*), and XPO1 (*X*) proteins shuttles through the nuclear pore complex (*NPC*) into the cytoplasm where RanGTP is dephosphorylated by the action of RanGAP, the Ran GTPase-activating protein. Cargo/XPO1 complexes are dissociated, and XPO1 shuttles back to the nucleus for further rounds of nuclear export

The XPO1 protein 3D conformation and XPO1-mediated nuclear export require the action of Ran (Ras-related nuclear protein), a small G protein. A RanGTP-RanGDP gradient is maintained across the nuclear membrane due to the subcellular localization of Ran regulators. Indeed, RanGDP is converted into RanGTP through the action of RCC1 (regulator of chromosome condensation 1), the Ran guanine nucleotide exchange factor, which is tethered to the chromatin. In contrast, RanGAP, the GTPase-activating protein, is cytosolic or bound to the outer cytoplasmic side of the nuclear pore complex (NPC). RanGAP allows the dephosphorylation of RanGTP into RanGDP. As shown in Fig. 1, RanGTP and cargos bind XPO1 in a cooperative manner, forming stable ternary complexes that are exported through the NPC. These complexes are disassembled in the cytoplasm, cargos are released, and XPO1 is recycled back to the nucleus for further rounds of export [24]. The association–dissociation of XPO1-cargo complexes are, thus, regulated by the direct binding of Ran in a compartment-specific manner.

**Fig. 2:**
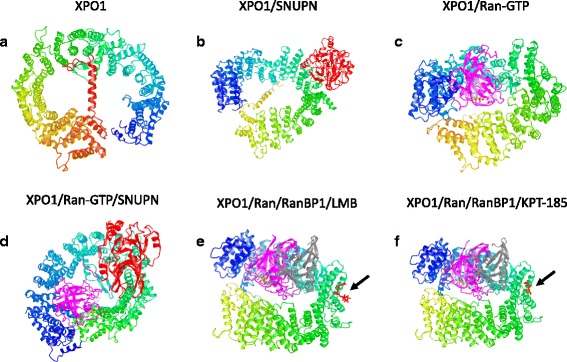
3D-XPO1 conformations illustrating its interaction with RanGTP, RanGTP-SNUPN, LMB, and KPT-285. (**a**) Ring-shaped XPO1 is colored *blue* to *red* in rainbow colors along the chain from the N-terminus to the C-terminus, showing the C-helix (*red*) autoinhibitory activity (PDB-ID 4FGV) [96], (**b**) XPO1 complexed with snurportin 1 (SNUPN) (PDB-ID 3GB8) [27], (**c**) with RanGTP, in *magenta* (PDB-ID 5DIS) [97], or (**d**) with both proteins (PDB-ID 3NC1) [28]. XPO1 inhibitors LMB (**e**) or KPT-185 (**f**) (both in *red* and *arrowed*) interacting with XPO1-RanGTP-RanBP1 (in *gray*) (PDB-ID, 4HAT [98], and 4GMX [9])

Structural analyses have shed light on the XPO1-mediated export molecular mechanism. Crystal structures of XPO1 bound to various NES-cargos and ternary complexes of RanGTP/XPO1/cargos are available in the Protein Data Bank [25]. Human XPO1 is a 120-kDa protein containing 21 consecutive tandem HEAT repeats (H1–H21). Each HEAT repeat is formed by two antiparallel α-helices A and B connected by loops of varying lengths. The A helices align to form an outer convex surface whereas the B helices form an inner concave surface (Fig. 2) [26, 27]. The N-terminal HEAT and the C-terminal HEAT are in close proximity, and XPO1 adopts a ring or toroidal shape. The A helices of H11 and H12 form a hydrophobic groove that constitutes the NES-binding site. The so-called leucine-rich NES motif, which is present in XPO1 cargo proteins, is a short peptide sequence of 10 ordered amino acid residues as follows: Φ1X2-3Φ2X2-3-Φ3XΦ4, where Φ is either isoleucine, leucine, methionine, phenylalanine, or valine, and X is any other amino acids [28]. The hydrophobic side chain of NES fits into five hydrophobic pockets along the NES-binding groove [26, 27, 29]. The NES-binding groove of the unliganded XPO1 adopts a closed conformation, which is stabilized by the binding of the H9 loop to the inner surface of H11 and H12. In this inhibited conformation, the B-helix of H21 (called the C-helix) lies within the central cavity with its C-terminus closed to the NES-binding site [30]. The transition from this inactive state to an active state is dependent on the binding of both RanGTP and cargos. RanGTP associates with the inner surface of XPO1 due to the movement of the H9 loop and C-helix away from the inner surface [31]. In the cytoplasm, the ternary XPO1-cargo-RanGTP complexes are dissociated. The binding of the Ran-binding domains (RanBDs) present in RanGAP and the associated proteins Ran-binding proteins 1 and 2 (RanBP1/2) induces a movement of the H9 loop, driving the rotation and translation of H11 and H12 and the release of cargos [31, 32]. Thus, the affinity of XPO1 to its cargos is drastically reduced by the site-directed mutagenesis of residues within the NES-binding groove [28, 29]. Conversely, mutations of the residues within the H9 loop or the deletion of the C-helix increases the affinity of XPO1 to its cargos [26, 32] and reduces the rate of cargo release [31]. Furthermore, García-Santisteban et al. recently described a new cellular reporter to investigate XPO1 nuclear export activity and the functional consequences of the highly recurrent cancer-related E571K mutation [33]. They showed that this mutation increases the affinity of the XPO1 protein to NES sequences, which may shift the nucleocytoplasmic transportation and thus modify the cell equilibrium; thus, the mutant *XPO1* E571K plays a role in carcinogenesis.

## XPO1 functions: nuclear export and mitosis control

As mentioned previously, NES sequences are diverse but conform to the consensus sequence Φ1X2-3Φ2X2-3-Φ3XΦ4. NESs were first described in the Rev protein of HIV and the cyclic AMP-dependent protein kinase inhibitor α (PKIα) [34, 35]. Currently, NESs have been identified in more than 200 proteins with various functions, such as cell cycle regulators, transcription factors, ribonucleoprotein complexes, translation factors, and viral proteins. NESs are compiled in the following comprehensive database: NESdb© [36, 37]. Among the XPO1 cargos, several proteins such as TSPs (p53, p73, FOXO1), growth regulators (RB, p21, CDC25, cyclin B1/D1), and anti-apoptotic molecules (nucleophosmin, survivin) control essential physiological cellular processes. Thus, correct XPO1 function is mandatory for normal cell homeostasis. In turn, abnormalities in nuclear export lead to diseases, including solid and hematologic cancers. In eukaryotes, RNAs are exported from the nucleus through the NPC. rRNAs (60S and 40S subunits), snRNAs, and a subset of miRNAs and mRNAs are exported in an XPO1-dependent manner [38, 39]. RNA export requires specific adaptor proteins, such as NDM3 [40].

Independently of its role in nuclear export, XPO1 is involved in the maintenance of chromosome integrity and the nucleation of microtubules at the kinetochore [41, 42]. With its partners (RanBP2, RanGAP, and RanGTP), XPO1 stabilizes the microtubules to the kinetochores, thereby allowing proper chromosome segregation [43]. XPO1 is also present at the centrosomes throughout the cell cycle. In complexes with RanGTP, XPO1 recruits pericentrin, the major centrosomal scaffold protein. Together with the microtubule nucleator ϒ-TuRC complexes, XPO1 permits spindle microtubule assembly [44]. Finally, RanGTP/XPO1 complexes bind nucleophosmin and maintain the fidelity of centrosome duplication [41, 44]. It is worth noting that the abnormal functioning of the dual XPO1 functions (nuclear export and mitosis regulation) could be highly deleterious for cells initiating and/or maintaining transformation processes.

## Somatic mutations in CLL, PMBL, cHL, and other B-cell malignancies

**Fig. 3:**
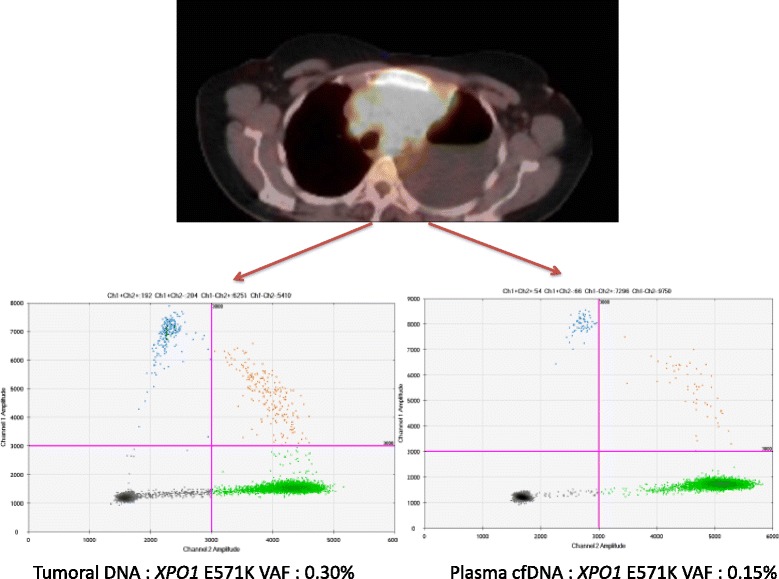
Positron emission tomography (PET) and digital PCR plots from a biopsy specimen and plasma cell-free DNA from a classical Hodgkin lymphoma patient with mediastinal involvement. Representative views of digital PCR (dPCR) plots (mutant samples) for *XPO1* E571K quantification in a tumor (VAF = 0.3%, *left*) and plasma cell-free DNA (cfDNA) (VAF = 0.15%, *right*) from a cHL patient at diagnosis by droplet dPCR (Biorad). Data are displayed in a scatter plot based on the color of FAM and VIC events. *Green*, *blue*, *orange*, and *black plots* represent wild-type alleles, mutant alleles, both alleles in the same droplet, or no amplifications, respectively. *VAF,* variant allele fraction

In a recent work based on NGS, the high prevalence of a recurrent single nucleotide variant (SNV) (E571K) of *XPO1* in both PMBL and cHL was demonstrated for the first time [12]. This mutation was observed in 25% of cases and appeared to be a specific genetic feature of these lymphomas as this mutation was observed at a very low frequency or was absent from mediastinal gray-zone lymphoma (MGZL) and GCB or activated B cell-like (ABC) DLBCL cases. In another recent study, recurrent *XPO1* E571K mutations were found in a large cohort of 94 patients with cHL using digital PCR (Fig. 3) and NGS experiments [16]. This novel information might provide new guidance on driver events and tumorigenesis in cHL. In total, 24% of patients with cHL harbored the *XPO1* E571K mutation. In this study, including 94 patients with all stage of cHL undergoing first-line treatment, the overall survival (OS) and progression-free survival (PFS) were similar between the mutated and wild-type patients at a median follow-up of 34.5 months. Furthermore, no alternative *XPO1* variants were detected by NGS on cHL biopsies; however, to date, the impact of the highly selected E571K mutation in the pathogenesis of cHL remains unknown. Several proteins known to play a major role in cHL oncogenesis, including STAT1, FOXO1, or CIITA, have also been identified as cargo proteins [45–47]. Whether the *XPO1* mutations impair the nucleus/cytoplasm transport of these proteins remains to be confirmed.

There is clear evidence for a pathological imbrication between PMBL and cHL, specifically the nodular sclerosis subtype; whether both might derive from thymic B cells is currently an area of discussion [48]. The fact that the XPO1 E571K mutation is enhanced in both these subtypes emphasizes the hypothesis of a common origin and a strong oncogenic role for this gene. The XPO1 mutation might serve as a distinctive genetic feature that facilitates the differential diagnosis of PMBL from DLBCL with mediastinal involvement, or MGZL, which are genetically similar but clearly distinct in their natural history and outcome [12, 49, 50].

Missense substitutions targeting *XPO1* have also been previously described at a low frequency (<5%) by NGS experiments in CLL [17, 19] and ESCC [18]. Notably, in a recent report, *XPO1* mutations were also detected in 38 of 486 (7.8%) CLL patients, 74% of which were E571K hotspot mutations [2]. An adverse prognostic impact was demonstrated with these *XPO1* mutations, but the overall outcome was not poor in patients receiving ibrutinib. Although the oncogenic process of mutations in patients with CLL, PMBL, and cHL are unclear, the recurrent nature of *XPO1* mutations in these malignancies strongly supports its involvement in the pathogenesis of the disease and suggests that these *XPO1* mutations may occur in various oncogenic mechanisms.

## Translocations and other mechanisms of expression deregulation and copy gains

The *XPO1* overexpression process in cancer cells is not currently clearly known. Several mechanisms may be involved, such as chromosomal translocations or gain of copies of the *XPO1* gene. It was recently demonstrated that a cryptic *XPO1*-*MLLT10* fusion detected by RNA-sequencing is associated with the deregulation of the *HOXA* gene locus expression [51]. *HOXA*-independent activity of the XPO1-AF10 fusion protein could contribute to leukemogenesis in T cell acute lymphoblastic leukemia (T-ALL) via an abnormal transport of tumor suppressors and growth-regulatory cellular factors and/or the dominant negative inhibition of wild-type *XPO1* [51].

Although the process of cyclin D1 nuclear import is insufficiently characterized in mantle cell lymphoma (MCL), the export of cyclin D1 complexes from the nucleus into the cytoplasm is established to be XPO1-dependent [52]. Indeed, the down-regulation of cyclin D1, which was accompanied by a substantial decrease of its target protein phospho-RB, was observed after a SINE agent KPT-185 treatment in MCL cells [53]. The t(11;14) nonrandom chromosomal translocation, leading to cyclin D1 overexpression that is typical of MCL disease, may also explain the *XPO1* overexpression in MCL cells. This hypothesis must be confirmed in dedicated studies to define the precise mechanisms of the deregulation of *XPO1*.

It has also been reported that XPO1 and other nuclear import receptors, such as importin-7, are regulated positively by *MYC* and negatively by p53 [54], resulting in the modulation of ribosomal biogenesis. *MYC* appears to transcriptionally upregulate *XPO1* as a part of a wide-ranging transcriptional program that also includes numerous ribosomal protein genes [55].

Recently, in 20 PMBL cases analyzed *via* CGH, a copy number gain in the *XPO1* locus was observed in eight cases [12]. This rate was higher than that observed for ABC DLBCL (8/70, 11%) but similar to that observed for GCB DLBCL (21/74, 28%). A significant correlation was observed between the *XPO1* copy number and the expression of the corresponding messenger RNA (mRNA), suggesting a gene dosage effect (*P* = 0.00106, Mann–Whitney test). Gains at the chromosome 2p16.1-2p15 locus, which contains both the *REL* and *XPO1* genes, were commonly observed in GCB DLBCL, cHL, and PMBL cases [56–58] and may be an important mechanism of *XPO1* overexpression in these hematological malignancies.

## *XPO1* mutations to assess minimal residual disease (MRD) in cHL and PMBL

The conveniences of using plasma cell-free DNA (cfDNA) for tumor mutation detection include (i) noninvasive acquisition, (ii) ability to be collected at any time during the disease course, (iii) real-time detection and follow-up of biomarker dynamics, and (iv) probably fewer heterogeneity concerns than tumor tissue testing [59]. Notably, the idea of a “liquid biopsy” was recently featured for the first time in a series of DLBCL patients, for whom high-throughput sequencing of a panel of target genes was implemented with an outstanding detection of somatic variants both in the tumor and the plasma [60]. Undeniably, the testing of mutations in the cfDNA has been widely characterized in many cancer types and could potentially serve as a biomarker tool for MRD, revealing treatment success or disease relapse before the clinico-radiological symptoms [61–64].

In contrast, Lymphosight® technology [65], which is based on the quantification of clonotypic immunoglobulin rearrangements, can be used more extensively to predict relapse in DLBCL patients [66], but a VDJ rearrangement is not measurable in all patients (immunoglobulin-negative phenotype of PMBL [67]), and the technology is not suitable for tailoring a targeted therapy. Furthermore, a recent proof-of-concept report exposed, for the first time, that the detection and measurement of recurrent somatic mutations in the plasma cfDNA of patients with DLBCL by digital PCR is possible, easy, and reproducible [68]. cfDNA testing by digital PCR (dPCR) is emerging as an appropriate and helpful molecular tool for the management of DLBCL along with NGS methods.

Finally, a recent retrospective study, including 94 all-stage cHL patients undergoing a first-line therapy, demonstrated that the highly recurrent *XPO1* E571K mutation is present in one-quarter of classical Hodgkin’s lymphoma patients [16]. Among these 94 patients, 50 had serial EDTA plasma samples obtained from blood collection concomitant with the diagnostic biopsy and at the end of chemotherapy/radiotherapy treatment. Patients with a detectable *XPO1* mutation in plasma cfDNA by digital PCR at the end of treatment exhibited a tendency toward shorter progression-free survival compared to patients with undetectable mutations. These results advocate that the clearance of the *XPO1* mutation in plasma cfDNA during and at the end of treatment may serve as a new prognostic marker for patients with detectable mutations. A study with a larger prospective cohort is needed to draw precise conclusions and determine whether this mutation monitoring adds new applicable value compared to positron emission tomography (PET) and whether this E571K variant can be targeted by SINE compounds for the treatment of cHL.

## XPO1 dysfunction as a diagnostic and/or prognostic marker

*XPO1* overexpression positively correlated with a larger tumor size in ESCC [18]. Moreover, the XPO1-mutated tumor also showed upregulated protein levels compared with the matched adjacent normal esophageal epithelium, indicating a gain-of-function phenotype. In hematological malignancies, high levels of *XPO1* are associated with shorter survival and are a poor prognostic factor in acute myeloid leukemia (AML) [69]. In this pathology, high XPO1 levels correlate with low MDM2 and high p53 levels.

Multiple myeloma (MM) cells have a higher XPO1 expression than bone marrow normal plasma cells and cells from monoclonal gammopathy of unknown significance (MGUS) patients [70].

An altered subcellular localization of XPO1 in MCL cell lines and primary cells and the knockdown of *XPO1* gene expression resulted in the inhibition of MCL cell growth [71]. *XPO1* is overexpressed in MCL cells and can regulate MCL cell proliferation, cell cycle progression, DNA damage response, and chromosomal stability, making it a promising therapeutic target for MCL management [71]. Another study confirmed these results and revealed that *XPO1* overexpression has a negative prognostic impact on MCL overall disease survival [72]. This work highlighted that the induction of p53 by SINE agents could potently induce cell death in MCL, including those with high levels of XPO1. The unfavorable prognostic impact of high levels of XPO1 expression was also demonstrated in an impressive study in MM [73]. In this work, high XPO1 expression in MM patient cells was associated with lytic bone disease and a shorter survival, and bortezomib-resistant patient MM cells expressed higher XPO1 levels. The authors showed that SINE treatment induced potent and rapid apoptosis of MM cells in vitro and in vivo and further directly decreased bone resorption. These results are interesting additional data supporting a pathogenic role of XPO1 in hematological malignancies and providing the pre-clinical rational for the ongoing clinical development of selinexor.

Nevertheless, cHL patients with tumors harboring the E571K *XPO1* mutation did not have a shorter PFS or OS than cLH patients with a wild-type *XPO1* gene [16]. By contrast the E571K *XPO1* mutation may have a﻿n unfavorable ﻿prognostic relevance for PMBL [12], which is in accordance with the observations reported for CLL [17].

## Targeting XPO1 by SINE compounds

**Fig. 4:**
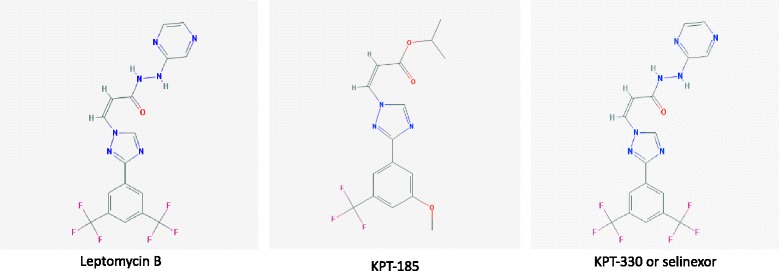
Chemical structures of leptomycin B and two SINE compounds. Chemical structures have been obtained from [99]

Leptomycin B (LMB), an anti-fungal antibiotic produced by *Streptomyces*, was the first described XPO1 inhibitor (Fig. 4). In their seminal report, Kudo and colleagues showed that LMB binds XPO1 at a conserved cysteine residue (position 528 in the human protein, C528) [74]. The alkylation of this cysteine residue, within the hydrophobic groove of XPO1, by LBM disrupts XPO1/cargo interactions and prevents the binding of RanGTP. Thus, LMB blocks nuclear export. Interestingly, the substitution C528S has no impact on XPO1 function, but cells carrying the C528S mutation are completely resistant to LMB. Although highly potent, even at nano-molar concentrations, LMB cannot be used in the clinic because of its high toxicity [75]. Recently, using computational methods, several XPO1 inhibitors, called selective inhibitors of nuclear export (SINE), have been developed by Karyopharm Therapeutics (e.g., KPT-185 and KPT-330, also known as selinexor, Fig. 4). SINEs inhibit the formation of XPO1/cargo complexes. Moreover, the crystal structures of SINEs showed that they bind covalently to C528, such as LMB [8, 9]. A homozygous mutation in the cysteine residue (C528S), using the CRISPR/Cas9 technology in the Jurkat T-ALL cell line, confers cell resistance to selinexor [76]. This result validates the specificity of the SINE to its target, XPO1. SINEs display anti-proliferative and pro-apoptotic activities in various hematological malignancies in both in vivo and in vitro settings, including cell lines and primary cells [15]. The main results of published pre-clinical studies are presented in Table 1. SINEs inhibit the XPO1 nuclear export function and restore the nuclear localization of TSPs and cell cycle proteins, thus restoring their functions. Importantly, SINEs target tumor cells but not normal hematopoietic cells [8, 9]. SINEs can also indirectly restore TSP and cell cycle regulator functions. In addition to the high XPO1 expression found in AML cells, the NPM1 gene encoding nucleophosmin 1 is mutated in up to 35% of cases [77]. These mutations cause increased XPO1 binding and the mislocalization of nucleophosmin 1 in the cytoplasm [78]. KPT-185 restores the nuclear localization of nucleophosmin 1, resulting in cell cycle arrest and AML blast differentiation [79].

**Table 1:** Pre-clinical in vitro and in vivo studies of KPT compounds on hematological malignancies

Disease	XPO1 gene and protein	SINE	Effects	Reference
AML		KPT-185	Induces apoptosis Promotes cell cycle arrest and inhibits proliferation Decreases XPO1 level and restores nuclear export Downregulates FLT3 and KIT oncogenes expression	[79]
		KPT-276	Increases survival of FLT3-ITD+-MV4.11 mice	
CLL	High XPO1 expression	KPT-185	Inhibits nuclear export Induces apoptosis of tumor cells not of normal B cells Targets the IκB pathway Antagonizes microenvironment stimuli (TNF, IL6, IL4) Inhibits tumor growth in vivo (Eμ-TCL1-SCID model)	[9]
T-ALL		KPT-185	Induce apoptosis in vitro and in vivo	[8]
		KPT-330	Promote cell cycle arrest in G1 Inhibit tumor growth in vivo	
AML	High XPO1 expression	KPT-185	Inhibits nuclear export Induces a p53-dependent cell apoptosis Inhibits cell proliferation in a p53-idependent manner	
AML		KPT-330	Inhibits XPO1/cargo interactions and nuclear export	[8, 100]
		KPT-251	Induces apoptosis Promotes cell cycle arrest in G1 Inhibits tumor growth in vivo Inhibits tumor growth in vivo	
MCL	High XPO1 expression	KPT-185	Blocks nuclear export	[71]
		KPT-276	Induces apoptosis in a p53-independent manner Has no effect on cell cycle Inactivates the NF-κB pathway Inhibits tumor growth in vivo	
MM	High XPO1 expression	KPT-276	Induces cell apoptosis in vitro and in vivo Targets c-Myc, CDC25A, and BRD4 Induces cell cycle arrest in G1	[70]
MM	High XPO1 expression	KPT-185	Inhibit nuclear export	[73]
		KPT-330	Induce apoposis and alleviate CAM-DR Promote cell cycle arrest in G1 Target c-Myc, MCL1, and NF-κB pathway Show a strong anti-myeloma activity in vivo, impair osteoclastogenesis and bone resorption	
NHL		KPT-185	Inhibits cell growth Induces apoptosis Restores the nuclear localization of TSPs and their function Shows anti-tumor activity in vivo	[101]
Ph + ALL	High XPO1 expression	KPT-330	Induces apoptosis both p53-dependent and -independent Decreases clonogenic potential Increases survival of BCR-ABL1 mice Alters the localization of hnRNP A1 and SET Reactivates the TSP PP2A	[102]
CLL		KPT-330	Suppresses effectors of BCR signaling in vitro and in vivo via BTK depletion Prevents CLL cells migration	[103]
HL	Mutation E571K XPO1 amplification	KPT-185	Inhibits cell line proliferation and induces apoptosis whatever XPO1 status	[16]
PMBL	Mutation E571K XPO1 amplification	KPT-185	Inhibits cell line proliferation and induces apoptosis whatever XPO1 status	[12]
AML/CLL/DLBCL		KPT-8602	Inhibits XPO1/cargo interactions and nuclear export Induces apoptosis of primary CLL cells Inhibits proliferation of DLBCL cell lines (ABC and GC subtypes) Prolongs the survival of Eμ-TCL1 mice Acts in synergy with ibrutinib in vivo Inhibits proliferation and induces apoptosis of AML cell lines and primary blasts Is efficient in a mouse model of AML	[85]

*Abbreviations*: *ABC* activated B cell like, *ALL* acute lymphoid leukemia, *AML* acute myeloid leukemia, *BCR* B cell receptor, BRD4 bromodomain-containing protein 4, *BTK* Bruton tyrosine kinase, *CAM-DR* cell adhesion-mediated drug resistance, *CDC25A* cell division cycle 25 homolog A, *CLL* chronic lymphoid leukemia, *DLBCL* diffuse large B cell lymphoma, *FLT3* FMS-like tyrosine kinase, *GC* germinal center, *IL* interleukin, *ITD* internal tandem duplication, *HL* Hodgkin lymphoma, hnRNP heterogeneous nuclear ribonucleoprotein, *MCL* mantle cell lymphoma, *MDM2* human homolog of mouse double minute 2, *MM* multiple myeloma, *NHL* non-Hodgkin lymphoma, *PP2A* protein phosphatase 2A, *PMBL* primary mediastinal B cell lymphoma, *TNF* tumor necrosis factor, *TSP* tumor suppressor protein

Pre-clinical studies in CLL cells with a mutated *XPO1* gene have not been reported thus far to our knowledge. However, in PMBCL and LH cell lines, KPT-185 inhibits cell proliferation and induces apoptosis, regardless of an amplified or mutated XPO1 gene status [12, 16].

Moreover, SINEs synergize with drugs used clinically to bypass the resistance that often occurs after treatment. In AML, topoisomerase (Topo) 2α (an XPO1 cargo) is aberrantly localized in the cytoplasm, leading to resistance to Topo2 inhibitors. Ranganathan and colleagues recently reported the synergistic activity of selinexor with Topo2 inhibitors (idarubicin, daunorubicin) in AML cell lines, primary AML blasts, and a murine xenograft model [80]. Mechanistically, selinexor restores the nuclear localization of Topo2, downregulates genes in the DNA damage response pathway and impairs homologous recombination for DNA repair. In AML, a high level of XPO1 correlates with a high level of p53 and a low level of mouse double minute 2 (MDM2, the negative regulator of p53). KPT-185 and Nutlin-3a, inhibitors of MDM2, display synergistic activity on apoptosis induction both in AML cells lines and primary cells [69]. Indeed, the nuclear accumulation of p53 induced by XPO1 inhibition is reinforced by the lack of p53 degradation resulting from MDM2 inhibition. Decitabine, an inhibitor of DNA methyltransferase that allows the re-expression of genes silenced during myeloid differentiation, appears to be efficient for some AML patients [81]. The pretreatment or priming of AML blasts with decitabine followed by selinexor enhances the induction of apoptosis in AML cell lines and primary AML blasts [82]. These effects are mediated by the re-expression of p21 and FOXO3A, which are XPO1 cargos that are silenced by the DNA methylation. In vivo, in a mouse model of AML, a selinexor/decitabine combination shows enhanced anti-leukemia activity.

CLL cells exhibit a constitutive activation of the BCR signaling pathway. Ibrutinib, which inhibits BTK (Bruton tyrosine kinase) expressed in CLL, is an effective therapy. However, acquired resistance to ibrutinib is frequent, partly due to a mutation in the BTK gene (C481S). The dual targeting of XPO1 by selinexor and BTK by ibrutinib elicits a synergistic effect both in vitro in primary cells and in vivo in the Eμ-TCL1 mouse model of CLL [83]. Importantly, the selinexor/ibrutinib combination bypasses the resistance due to the C481S BTK mutation.

The combination of an XPO1 inhibitor and liposomal doxorubicin appears highly effective in in vitro resistant MM models, xenograft studies, and ex vivo samples from patients with relapsed/refractory myeloma. In pre-clinical models, the anti-lymphoma activity of selinexor is enhanced through a combination with dexamethasone and everolimus, which target both NF-κB and mTOR [14]. A synergistic effect has been demonstrated with proteasome inhibitors (PIs), such as carfilzomib and bortezomib [84] and selinexor, the latter of which overcomes PI drug resistance in MM. This synergistic effect is mediated by blocking the phosphorylation of the IκB-α and the NF-κB p65 subunits, protecting IκB-α from proteasome degradation [13].

Although selinexor has shown its efficacy in phase I/II clinical trials (see below), its toxicity limits its administration. A new generation of SINE compounds has been designed by Karyopharm Therapeutics and assayed for efficacy in CLL and AML [85]. KPT-8602 acts similarly to selinexor; it inhibits XPO1/cargo interactions, induces apoptosis, and inhibits proliferation of primary CLL cells, AML cell lines, and AML blasts. In vivo, in mouse models of AML and CLL, KPT-8602 prolongs survival. More importantly, compared to selinexor, KPT-8602 appears to be less toxic because it has a reduced capacity to cross the blood–brain barrier and does not accumulate in the blood even after repetitive injections.

Resistance to therapy and relapse occur because of the inability of the currently used drugs to target the cancer-initiating cells (CICs) or leukemia-initiating cells (LICs). A CIC/LIC is defined as a cell, within the tumor population, that is able to self-renew, differentiate, and reproduce a tumor. Such an LIC has been described in AML [86, 87]. Selinexor appears to be active against primary AML cells, including LICs engrafted into immunodeficient mice. Moreover, selinexor shows limited toxicity against normal hematopoietic stem cells and progenitors [88].

The inhibition of RNA export and the impairment of ribosome biogenesis induce the death of MCL and MM. In MCL cells, KPT-185 induces a down-regulation of proliferation-related genes regardless of the status of p53 and, in turn, the inhibition of the cell cycle. Moreover, KPT-185 downregulates the expression of ribosomal proteins (from both the large 60S and the small 40S subunits). This affects heat shock factors, protein synthesis, and energy metabolism that are important for tumor cell proliferation and survival [53]. In MM cells, selinexor targets the RPL11 and RPL5 ribosomal proteins, decreases the 40S, 60S, and 80S ribosomal fractions, and disrupts the ribosomal function and translational machinery. This induced ribosomal stress leads to MM cell death [53].

XPO1 inhibition also represents a new therapeutic strategy for overcoming resistance to platinum-mediated apoptosis through both p53-dependent and p53-independent pathways [89]. The combination synergistically induced Topo 2α-mediated DNA damage and subsequent apoptosis. In solid tumor models, it has been demonstrated that selinexor acts synergistically with gemcitabine to promote apoptosis and reduce survival [90] and can enhance radiotherapy effects, providing a rational for such a combination in B cell malignancies [91], including non-Hodgkin lymphoma (NHL) and cHL.

Finally, in a model of synergistic colon/melanoma cancer, a combination of selinexor + programmed cell death 1 (PD-1 or PD-L1) blockade exerts considerable anti-tumor activity and shows significant immunomodulatory activity, inducing changes in the frequency and phenotype of immune cell populations, most notably in NK cells and activated T cells. These results support the future clinical evaluation of the combination of selinexor + PD-1/PD-L1 blockade, a strategy especially relevant in cHL [92]. Importantly, in vitro and in vivo experiments are providing the rational for the ongoing and future clinical trials based on the combination of XPO1 inhibitors and approved drugs (Table 2).

**Table 2:** Clinical trials in lymphoid malignancies testing selinexor as single agent or in combination

NCT	Phase	Lymphoid malignancy	Therapeutic strategy/combination	Estimated enrollment	Status
NCT01607892	I	Hematologic malignancies	Selinexor	285	Not recruiting
NCT02138786	II	Richter’s transformation lymphoma	Selinexor	26	Terminated
NCT02186834	I/II	MM	Selinexor, liposomal doxorubicin, dexamethasone	47	Recruiting
NCT02199665	I	MM	Selinexor, carfilzomib, dexamethasone	48	Recruiting
NCT02227251	II	R/R DLBCL	Selinexor	200	Recruiting
NCT02303392	I	CLL, lymphoma	Selinexor, ibrutinib	92	Recruiting
NCT02314247	II	TCL	Selinexor	16	Terminated
NCT02336815	II	MM	Selinexor, dexamethasone	210	Recruiting
NCT02343042	I/II	MM	Selinexor, dexamethasone, bortezomib, pomalidomide	201	Recruiting
NCT02389543	I/II	MM	Selinexor, lenalidomide, dexamethasone	34	Withdrawn
NCT02471911	I	R/R aggressive B cell lymphoma	Selinexor, rituximab, etoposide, carboplatin, ifosfamide, dexamethasone	18	Recruiting
NCT02741388	I	R/R B cell lymphoma	Selinexor, rituximab, dexamethasone, oxaliplatin, cisplatin, cytarabine, gemcitabine	60	Recruiting

Data are from ClinicalTrials.gov [104]

*Abbreviations*: *CLL* chronic lymphoid leukemia, *MM* multiple myeloma, *R/R* refractory/relapse, *TCL* T-cell lymphoid leukemia

These data reinforce the concept of XPO1 as an anti-cancer target and SINEs as efficient anti-XPO1 drugs in human hematological malignancies.

## Clinical experience in B-cell malignancies

Giving the pleiotropic and crucial role of XPO1 and the fact that the overexpression of XPO1 has been associated with resistance and aggressiveness in multiple tumor types, several clinical trials using drugs targeting XPO1 are currently ongoing in the field of hematological malignancies and solid tumors. The ongoing or terminated clinical trials testing the SINE selinexor compound in lymphoid malignancies are summarized in Table 2. Consolidated clinical results in CLL, MM, and NHL are still limited.

In a phase 1 dose-escalation study of selinexor in patients with heavily pre-treated NHL, among the 43 evaluable patients, an overall response rate of 28% was observed with a complete response rate of 5%. In the DLBCL cohort of 39 evaluable patients, the disease control rate was 51%, with overall response rate of 31%. Interestingly in DLBCL patients with MYC/BCL2 translocations, three of the four patients showed an objective response [93].

The toxicity profile of selinexor used as a single agent is currently well-known. In a phase 1 trial involving 189 patients with advanced solid tumors receiving selinexor, the most common treatment-related adverse events included fatigue (70%), nausea (70%), anorexia (66%), and vomiting (49%), which were generally grades 1 or 2. The most commonly reported grade 3 or 4 toxicities were thrombocytopenia (16%), fatigue (15%), and hyponatremia (13%). Clinically significant major organ or cumulative toxicities were rare. The maximum-tolerated dose was set at 65 mg/m^2^ using a twice-a-week (days 1 and 3) dosing schedule [11]. Selinexor was significantly better tolerated when administered as a flat dose on an intermittent schedule. Pharmacokinetics analysis of selinexor revealed an increase (approximately 15 to 20%) in drug exposure when taken with food [94]. A phase I trial evaluated the combination of selinexor with fludarabine and cytarabine in pediatric R/R acute leukemia. The most common grade 3 non-hematologic toxicity was asymptomatic hyponatremia. Two patients who were treated at 70 mg/m^2^ experienced reversible cerebellar toxicity, thereby defining the dose-limiting toxicity [95].

Interestingly, the new SINE compound KPT-8602 shows similar in vitro potency compared with selinexor but lower central nervous system penetration, which resulted in enhanced tolerability, even when dosed daily, and improved survival in CLL and AML murine models [80, 82].

## Conclusion

XPO1 represents an exciting field located at the intersection between pathophysiology, diagnosis, and treatment of B cell malignancies. Many crucial points and burning questions need to be addressed. At the biological level, the respective role of XPO1 in distinct B/T cell malignancies is still largely unknown, and an accurate view of the cargo molecules (including proteins and RNAs) is still lacking. At the genetic level, the highly selected recurrent E571K mutation does not appear to impact the activity of SINE compounds, but its biological relevance, especially in cHL and PMBL, is still undetermined. Interestingly, this hot spot may be used as a simple MRD marker to complement PET scan imaging in these diseases. Finally, from a clinical point of view, blocking XPO1 activity represents a promising strategy in lymphoid malignancies and has synergistic effects with several drugs, including anthracyclines, gemcitabine, mTOR inhibitors, ibrutinib, immune check-points inhibitors or radiotherapy.

## Abbreviations

ABC: Activated B-cell like
AML: Acute myeloid leukemia
APC: Adenomatous polyposis coli
BCR: B-cell receptor
BD: Binding domain
BRCA1: Breast cancer 1
BRD4: Bromodomain-containing protein 4
BTK: Bruton tyrosine kinase
CAM-DR: Cell adhesion-mediated drug resistance
CDC25A: Cell division cycle 25 homolog A
cfDNA: Cell-free DNA
CGH: Comparative genomic hybridization
cHL: Classical Hodgkin lymphoma
CIC: Cancer-initiating cell
CLL: Chronic lymphoid leukemia
CRM1: Chromosome region maintenance 1
DLBCL: Diffuse large B cell lymphoma
dPCR: Digital PCR
ESCC: Esophageal squamous cell carcinoma
FLT3: FMS-like tyrosine kinase
FOXO: Forkhead box class O
GC: Germinal center
GCB: Germinal B-cell-like
hnRNP: Heterogeneous nuclear ribonucleoprotein
IL: Interleukin
ITD: Internal tandem duplication
LIC: Leukemia-initiating cell
LMB: Leptomycin B
MCL: Mantle cell lymphoma
MDM2: Mouse double minute 2
MGUS: Monoclonal gammopathy of undetermined significance
MGZL: Mediastinal gray-zone lymphoma
MM: Multiple myeloma
MRD: Minimal residual disease
mTOR: Mammalian target of rapamycin
NES: Nuclear export signal
NGS: Next-generation sequencing
NHL: Non-Hodgkin lymphoma
NPC: Nuclear pore complex
PET: Positron emission tomography
PMBL: Primary mediastinal B cell lymphoma
PP2A: Protein phosphatase 2A
RB: Retinoblastoma protein
SINE: small inhibitor of nuclear export
SNV: Single-nucleotide variant
STAT: Signal transducer and activator of transcription
T-ALL: T-cell acute lymphoid leukemia
TNF: Tumor necrosis factor
Topo: Topoisomerase
TSP: Tumor suppressor protein
XPO1: Exportin 1

## References

[1] Huang WY, Yue L, Qiu WS, Wang LW, Zhou X-H, Sun YJ (2009). Prognostic value of CRM1 in pancreas cancer. Clin Invest Med..

[2] Jain P, Kanagal-Shamanna R, Wierda W, Keating M, Sarwari N, Rozovski U, Thompson P, Burger J, Kantarjian H, Patel KP, Medeiros LJ, Luthra R, Estrov Z (2016). Clinical and molecular characteristics of XPO1 mutations in patients with chronic lymphocytic leukemia. Am J Hematol..

[3] Kim J, McMillan E, Kim HS, Venkateswaran N, Makkar G, Rodriguez-Canales J, Villalobos P, Neggers JE, Mendiratta S, Wei S, Landesman Y, Senapedis W, Baloglu E, Chow CB, Frink RE, Gao B, Roth M, Minna JD, Daelemans D, Wistuba II, Posner BA, Scaglioni PP, White MA (2016). XPO1-dependent nuclear export is a druggable vulnerability in KRAS-mutant lung cancer. Nature..

[4] Liu X, Chong Y, Tu Y, Liu N, Yue C, Qi Z, Liu H, Yao Y, Liu H, Gao S, Niu M, Yu R (2016). CRM1/XPO1 is associated with clinical outcome in glioma and represents a therapeutic target by perturbing multiple core pathways. J Hematol Oncol..

[5] Noske A, Weichert W, Niesporek S, Röske A, Buckendahl A-C, Koch I, Sehouli J, Dietel M, Denkert C (2008). Expression of the nuclear export protein chromosomal region maintenance/exportin 1/Xpo1 is a prognostic factor in human ovarian cancer. Cancer..

[6] van der Watt P, Zemanay W, Govender D, Hendricks D, Parker M, Leaner V (2014). Elevated expression of the nuclear export protein, Crm1 (exportin 1), associates with human oesophageal squamous cell carcinoma. Oncol Rep..

[7] Yao Y, Dong Y, Lin F, Zhao H, Shen Z, Chen P, Sun YJ, Tang LN, Zheng SE (2009). The expression of CRM1 is associated with prognosis in human osteosarcoma. Oncol Rep..

[8] Etchin J, Sun Q, Kentsis A, Farmer A, Zhang ZC, Sanda T, Mansour MR, Barcelo C, McCauley D, Kauffman M, Shacham S, Christie AL, Kung AL, Rodig SJ, Chook YM, Look AT (2013). Antileukemic activity of nuclear export inhibitors that spare normal hematopoietic cells. Leukemia..

[9] Lapalombella R, Sun Q, Williams K, Tangeman L, Jha S, Zhong Y, Goettl V, Mahoney E, Berglund C, Gupta S, Farmer A, Mani R, Johnson AJ, Lucas D, Mo X, Daelemans D, Sandanayaka V, Shechter S, McCauley D, Shacham S, Kauffman M, Chook YM, Byrd JC (2012). Selective inhibitors of nuclear export show that CRM1/XPO1 is a target in chronic lymphocytic leukemia. Blood..

[10] Van Neck T, Pannecouque C, Vanstreels E, Stevens M, Dehaen W, Daelemans D (2008). Inhibition of the CRM1-mediated nucleocytoplasmic transport by N-azolylacrylates: structure-activity relationship and mechanism of action. Bioorg Med Chem..

[11] Abdul Razak AR, Mau-Soerensen M, Gabrail NY, Gerecitano JF, Shields AF, Unger TJ, Saint-Martin JR, Carlson R, Landesman Y, McCauley D, Rashal T, Lassen U, Kim R, Stayner LA, Mirza MR, Kauffman M, Shacham S, Mahipal A (2016). First-in-class, first-in-human phase I study of selinexor, a selective inhibitor of nuclear export, in patients with advanced solid tumors. J Clin Oncol..

[12] Jardin F, Pujals A, Pelletier L, Bohers E, Camus V, Mareschal S, Dubois S, Sola B, Ochmann M, Lemonnier F, Viailly PJ, Bertrand P, Maingonnat C, Traverse-Glehen A, Gaulard P, Damotte D, Delarue R, Haioun C, Argueta C, Landesman Y, Salles G, Jais JP, Figeac M, Copie-Bergman C, Molina TJ, Picquenot JM, Cornic M, Fest T, Milpied N, Lemasle E, Stamatoullas A, Moeller P, Dyer MJ, Sundstrom C, Bastard C, Tilly H, Leroy K (2016). Recurrent mutations of the exportin 1 gene (XPO1) and their impact on selective inhibitor of nuclear export compounds sensitivity in primary mediastinal B-cell lymphoma. Am J Hematol.

[13] Kashyap T, Argueta C, Aboukameel A, Unger TJ, Klebanov B, Mohammad RM, Muqbil I, Azmi AS, Drolen C, Senapedis W, Lee M, Kauffman M, Shacham S, Landesman Y (2016). Selinexor, a selective inhibitor of nuclear export (SINE) compound, acts through NF-κB deactivation and combines with proteasome inhibitors to synergistically induce tumor cell death. Oncotarget..

[14] Muqbil I, Aboukameel A, Elloul S, Carlson R, Senapedis W, Baloglu E, Kauffman M, Shacham S, Bhutani D, Zonder J, Azmi AS, Mohammad RM (2016). Anti-tumor activity of selective inhibitor of nuclear export (SINE) compounds, is enhanced in non-Hodgkin lymphoma through combination with mTOR inhibitor and dexamethasone. Cancer Lett..

[15] Das A, Wei G, Parikh K, Liu D (2015). Selective inhibitors of nuclear export (SINE) in hematological malignancies. Exp Hematol Oncol..

[16] Camus V, Stamatoullas A, Mareschal S, Viailly PJ, Sarafan-Vasseur N, Bohers E, Dubois S, Picquenot JM, Ruminy P, Maingonnat C, Bertrand P, Cornic M, Tallon-Simon V, Becker S, Veresezan L, Frebourg T, Vera P, Bastard C, Tilly H, Jardin F (2016). Detection and prognostic value of recurrent exportin 1 mutations in tumor and cell-free circulating DNA of patients with classical Hodgkin lymphoma. Haematologica..

[17] Jeromin S, Weissmann S, Haferlach C, Dicker F, Bayer K, Grossmann V, Alpermann T, Roller A, Kohlmann A, Haferlach T, Kern W, Schnittger S (2014). SF3B1 mutations correlated to cytogenetics and mutations in NOTCH1, FBXW7, MYD88, XPO1 and TP53 in 1160 untreated CLL patients. Leukemia..

[18] Lin D-C, Hao J-J, Nagata Y, Xu L, Shang L, Meng X, Sato Y, Okuno Y, Varela AM, Ding LW, Garg M, Liu LZ, Yang H, Yin D, Shi ZZ, Jiang YY, Gu WY, Gong T, Zhang Y, Xu X, Kalid O, Shacham S, Ogawa S, Wang MR, Koeffler HP (2014). Genomic and molecular characterization of esophageal squamous cell carcinoma. Nat Genet..

[19] Puente XS, Pinyol M, Quesada V, Conde L, Ordóñez GR, Villamor N, Escaramis G, Jares P, Beà S, González-Díaz M, Bassaganyas L, Baumann T, Juan M, López-Guerra M, Colomer D, Tubío JM, López C, Navarro A, Tornador C, Aymerich M, Rozman M, Hernández JM, Puente DA, Freije JM, Velasco G, Gutiérrez-Fernández A, Costa D, Carrió A, Guijarro S, Enjuanes A, Hernández L, Yagüe J, Nicolás P, Romeo-Casabona CM, Himmelbauer H, Castillo E, Dohm JC, de Sanjosé S, Piris MA, de Alava E, San Miguel J, Royo R, Gelpí JL, Torrents D, Orozco M, Pisano DG, Valencia A, Guigó R, Bayés M, Heath S, Gut M, Klatt P, Marshall J, Raine K, Stebbings LA, Futreal PA, Stratton MR, Campbell PJ, Gut I, López-Guillermo A, Estivill X, Montserrat E, López-Otín C, Campo E (2011). Whole-genome sequencing identifies recurrent mutations in chronic lymphocytic leukaemia. Nature.

[20] Adachi Y, Yanagida M (1989). Higher order chromosome structure is affected by cold-sensitive mutations in a *Schizosaccharomyces pombe* gene *crm*1^+^ which encodes a 115-kD protein preferentially localized in the nucleus and at its periphery. J Cell Biol..

[21] Fornerod M, Ohno M, Yoshida M, Mattaj IW (1997). CRM1 Is an export receptor for leucine-rich nuclear export signals. Cell..

[22] Fukuda M, Asano S, Nakamura T, Adachi M, Yoshida M, Nishida E (1997). CRM1 is responsible for intracellular transport mediated by the nuclear export signal. Nature..

[23] Ossareh-Nazari B, Bachelerie FO, Dargemont C (1997). Evidence for a role of CRM1 in signal-mediated nuclear protein export. Science..

[24] Ishizawa J, Kojima K, Hail N, Tabe Y, Andreeff M (2015). Expression, function, and targeting of the nuclear exporter chromosome region maintenance 1 (CRM1) protein. Pharmacol Ther..

[25] RCSB Protein Data Bank. http://www.rcsb.org/pdb.

[26] Dong X, Biswas A, Süel KE, Jackson LK, Martinez R, Gu H, Chook YM (2009). Structural basis for leucine-rich nuclear export signal recognition by CRM1. Nature..

[27] Monecke T, Guttler T, Neumann P, Dickmanns A, Gorlich D, Ficner R (2009). Crystal Structure of the nuclear export receptor CRM1 in complex with snurportin1 and RanGTP. Science..

[28] Guttler T, Madl T, Neumann P, Deichsel D, Corsini L, Monecke T, Ficner R, Sattler M, Görlich D (2010). NES consensus redefined by structures of PKI-type and Rev-type nuclear export signals bound to CRM1. Nat Struct Mol Biol..

[29] Dong X, Biswas A, Chook YM (2009). Structural basis for assembly and disassembly of the CRM1 nuclear export complex. Nat Struct Mol Biol..

[30] Saito N, Matsuura Y (2013). A 2.1-Å-resolution crystal structure of unliganded CRM1 reveals the mechanism of autoinhibition. J Mol Biol.

[31] Koyama M, Matsuura Y (2010). An allosteric mechanism to displace nuclear export cargo from CRM1 and RanGTP by RanBP1. EMBO J..

[32] Fox AM, Ciziene D, McLaughlin SH, Stewart M (2011). Electrostatic interactions involving the extreme C terminus of nuclear export factor CRM1 modulate its affinity for cargo. J Biol Chem..

[33] García-Santisteban I, Arregi I, Alonso-Mariño M, Urbaneja MA, Garcia-Vallejo JJ, Bañuelos S, Rodríguez JA (2016). A cellular reporter to evaluate CRM1 nuclear export activity: functional analysis of the cancer-related mutant E571K. Cell Mol Life Sci..

[34] Fischer U, Huber J, Boelens WC, Mattaj IW, Luhrmann R (1995). The HIV-1 Rev activation domain is a nuclear export signal that accesses an export pathway used by specific cellular RNAs. Cell..

[35] Wen W, Meinkoth JL, Tsien RY, Taylor SS (1995). Identification of a signal for rapid export of proteins from the nucleus. Cell..

[36] Xu D, Grishin NV, Chook YM (2012). NESdb: a database of NES-containing CRM1 cargoes. Mol Biol Cell..

[37] Xu D, Farmer A, Collett G, Grishin NV, Chook YM (2012). Sequence and structural analyses of nuclear export signals in the NESdb database. Mol Biol Cell..

[38] Muqbil I, Bao B, Abou-Samra AB, Mohammad RM, Azmi AS (2014). Nuclear export mediated regulation of microRNAs: potential target for drug intervention. Curr Drug Targets..

[39] Okamura M, Inose H, Masuda S (2015). RNA export through the NPC in eukaryotes. Genes..

[40] Bai B, Moore HM, Laiho M (2013). CRM1 and its ribosome export adaptor NMD3 localize to the nucleolus and affect rRNA synthesis. Nucleus..

[41] Forbes DJ, Travesa A, Nord MS, Bernis C (2015). Nuclear transport factors: global regulation of mitosis. Curr Opin Cell Biol..

[42] Takeda A, Yaseen NR (2014). Nucleoporins and nucleocytoplasmic transport in hematologic malignancies. Sem Cancer Biol..

[43] Arnaoutov A, Dasso M (2006). Ran-GTP regulates kinetochore attachment in somatic cells. Cell Cycle..

[44] Liu Q, Jiang Q, Zhang C, Liu Q, Jiang Q, Zhang C (2009). A fraction of Crm1 locates at centrosomes by its CRIME domain and regulates the centrosomal localization of pericentrin. Biochem Biophys Res Commun..

[45] Chiu E, Gold T, Fettig V, LeVasseur MT, Cressman DE (2015). Identification of a nuclear export sequence in the MHC CIITA. J Immunol..

[46] Hao Y, Chapuy B, Monti S, Sun HH, Rodig SJ, Shipp MA (2014). Selective JAK2 inhibition specifically decreases Hodgkin lymphoma and mediastinal large B-cell lymphoma growth in vitro and in vivo. Clin Cancer Res..

[47] Xie L, Ushmorov A, Leithäuser F, Guan H, Steidl C, Färbinger J, Pelzer C, Vogel MJ, Maier HJ, Gascoyne RD, Möller P, Wirth T (2012). FOXO1 is a tumor suppressor in classical Hodgkin lymphoma. Blood..

[48] Traverse-Glehen A, Pittaluga S, Gaulard P, Sorbara L, Alonso MA, Raffeld M, Jaffe ES (2005). Mediastinal gray zone lymphoma: the missing link between classic Hodgkin’s lymphoma and mediastinal large B-cell lymphoma. Am J Surg Pathol..

[49] Dunleavy K, Wilson WH (2015). Primary mediastinal B-cell lymphoma and mediastinal gray zone lymphoma: do they require a unique therapeutic approach?. Blood..

[50] Dunleavy K, Grant C, Eberle FC, Pittaluga S, Jaffe ES, Wilson WH (2012). Gray zone lymphoma: better treated like Hodgkin lymphoma or mediastinal large B-cell lymphoma?. Curr Hematol Malig Rep..

[51] Bond J, Bergon A, Durand A, Tigaud I, Thomas X, Asnafi V, Spicuglia S, Macintyre E (2005). Cryptic *XPO1-MLLT10* translocation is associated with *HOXA* locus deregulation in T-ALL. Blood..

[52] Benzeno S, Diehl JA (2004). C-terminal sequences direct cyclin D1-CRM1 binding. J Biol Chem..

[53] Tabe Y, Kojima K, Yamamoto S, Sekihara K, Matsushita H, Davis RE, Wang Z, Ma W, Ishizawa J, Kazuno S, Kauffman M, Shacham S, Fujimura T, Ueno T, Miida T, Andreeff M (2015). Ribosomal biogenesis and translational flux inhibition by the selective inhibitor of nuclear export (SINE) XPO1 antagonist KPT-185. PLoS One..

[54] Golomb L, Bublik DR, Wilder S, Nevo R, Kiss V, Grabusic K, Volarevic S, Oren M (2012). Importin 7 and exportin 1 link c-Myc and p53 to regulation of ribosomal biogenesis. Mol Cell..

[55] Wu CH, Sahoo D, Arvanitis C, Bradon N, Dill DL, Felsher DW (2008). Combined analysis of murine and human microarrays and ChIP analysis reveals genes associated with the ability of MYC to maintain tumorigenesis. PLoS Genet..

[56] Lenz G, Wright GW, Emre NCT, Kohlhammer H, Dave SS, Davis RE, Carty S, Lam LT, Shaffer AL, Xiao W, Powell J, Rosenwald A, Ott G, Muller-Hermelink HK, Gascoyne RD, Connors JM, Campo E, Jaffe ES, Delabie J, Smeland EB, Rimsza LM, Fisher RI, Weisenburger DD, Chan WC, Staudt LM (2008). Molecular subtypes of diffuse large B-cell lymphoma arise by distinct genetic pathways. Proc Natl Acad Sci USA.

[57] Reichel J, Chadburn A, Rubinstein PG, Giulino-Roth L, Tam W, Liu Y, Gaiolla R, Eng K, Brody J, Inghirami G, Carlo-Stella C, Santoro A, Rahal D, Totonchy J, Elemento O, Cesarman E, Roshal M (2015). Flow sorting and exome sequencing reveal the oncogenome of primary Hodgkin and Reed-Sternberg cells. Blood..

[58] Weniger MA, Gesk S, Ehrlich S, Martin-Subero JI, Dyer MJS, Siebert R, Möller P, Barth TF (2007). Gains of REL in primary mediastinal B-cell lymphoma coincide with nuclear accumulation of REL protein. Genes Chromosomes Cancer..

[59] Diaz LA, Bardelli A (2014). Liquid biopsies: genotyping circulating tumor DNA. J Clin Oncol..

[60] Bohers E, Viailly PJ, Dubois S, Bertrand P, Maingonnat C, Mareschal S, Ruminy P, Picquenot JM, Bastard C, Desmots F, Fest T, Leroy K, Tilly H, Jardin F (2015). Somatic mutation of cell-free circulating DNA detected by next-generation sequencing reflect the genetic changes in both germinal center B-cell-like and activated B-cell-like diffuse large B-cell lymphomas at the time of diagnosis. Haematologica..

[61] Frattini M, Gallino G, Signoroni S, Balestra D, Battaglia L, Sozzi G, Leo E, Pilotti S, Pierotti MA (2006). Quantitative analysis of plasma DNA in colorectal cancer patients: a novel prognostic tool. Ann NY Acad Sci..

[62] Jung K, Stephan C, Lewandowski M, Klotzek S, Jung M, Kristiansen G, Lein M, Loening SA, Schnorr D (2004). Increased cell-free DNA in plasma of patients with metastatic spread in prostate cancer. Cancer Lett..

[63] Sozzi G, Conte D, Leon M, Ciricione R, Roz L, Ratcliffe C, Roz E, Cirenei N, Bellomi M, Pelosi G, Pierotti MA, Pastorino U (2003). Quantification of free circulating DNA as a diagnostic marker in lung cancer. J Clin Oncol..

[64] Leon SA, Shapiro B, Sklaroff DM, Yaros MJ (1977). Free DNA in the serum of cancer patients and the effect of therapy. Cancer Res..

[65] Kurtz DM, Green MR, Bratman SV, Scherer F, Liu CL, Kunder CA, Takahashi K, Glover C, Keane C, Kihira S, Visser B, Callahan J, Kong KA, Faham M, Corbelli KS, Miklos D, Advani RH, Levy R, Hicks RJ, Hertzberg M, Ohgami RS, Gandhi MK, Diehn M, Alizadeh AA (2015). Noninvasive monitoring of diffuse large B-cell lymphoma by immunoglobulin high-throughput sequencing. Blood..

[66] Roschewski M, Dunleavy K, Pittaluga S, Moorhead M, Pepin F, Kong K, Shovlin M, Jaffe ES, Staudt LM, Lai C, Steinberg SM, Chen CC, Zheng J, Willis TD, Faham M, Wilson WH (2015). Circulating tumour DNA and CT monitoring in patients with untreated diffuse large B-cell lymphoma: a correlative biomarker study. Lancet Oncol..

[67] Leithäuser F, Bäuerle M, Huynh MQ, Möller P (2001). Isotype-switched immunoglobulin genes with a high load of somatic hypermutation and lack of ongoing mutational activity are prevalent in mediastinal B-cell lymphoma. Blood..

[68] Camus V, Sarafan-Vasseur N, Bohers E, Dubois S, Mareschal S, Bertrand P, Viailly PJ, Ruminy P, Maingonnat C, Lemasle E, Stamatoullas A, Picquenot JM, Cornic M, Beaussire L, Bastard C, Frebourg T, Tilly H, Jardin F (2016). Digital PCR for quantification of recurrent and potentially actionable somatic mutations in circulating free DNA from patients with diffuse large B-cell lymphoma. Leuk Lymphoma..

[69] Kojima K, Kornblau SM, Ruvolo V, Dilip A, Duvvuri S, Davis RE, Zhang M, Wang Z, Coombes KR, Zhang N, Qiu YH, Burks JK, Kantarjian H, Shacham S, Kauffman M, Andreeff M (2013). Prognostic impact and targeting of CRM1 in acute myeloid leukemia. Blood..

[70] Schmidt J, Braggio E, Kortuem KM, Egan JB, Zhu YX, Xin CS, Tiedemann RE, Palmer SE, Garbitt VM, McCauley D, Kauffman M, Shacham S, Chesi M, Bergsagel PL, Stewart AK (2013). Genome-wide studies in multiple myeloma identify XPO1/CRM1as a critical target validated using the selective nuclear export inhibitor KPT-276. Leukemia..

[71] Zhang K, Wang M, Tamayo AT, Shacham S, Kauffman M, Lee J, Zhang L, Ou Z, Li C, Sun L, Ford RJ, Pham LV (2013). Novel selective inhibitors of nuclear export CRM1 antagonists for therapy in mantle cell lymphoma. Exp Hematol.

[72] Yoshimura M, Ishizawa J, Ruvolo V, Dilip A, Quintas-Cardama A, McDonnell TJ, Neelapu SS, Kwak LW, Shacham S, Kauffman M, Tabe Y, Yokoo M, Kimura S, Andreeff M, Kojima K (2014). Induction of p53-mediated transcription and apoptosis by exportin-1 (XPO1) inhibition in mantle cell lymphoma. Cancer Sci..

[73] Tai YT, Landesman Y, Acharya C, Calle Y, Zhong MY, Cea M, Tannenbaum D, Cagnetta A, Reagan M, Munshi AA, Senapedis W, Saint-Martin JR, Kashyap T, Shacham S, Kauffman M, Gu Y, Wu L, Ghobrial I, Zhan F, Kung AL, Schey SA, Richardson P, Munshi NC, Anderson KC (2014). CRM1 inhibition induces tumor cell cytotoxicity and impairs osteoclastogenesis in multiple myeloma: molecular mechanisms and therapeutic implications. Leukemia..

[74] Kudo N, Matsumori N, Taoka H, Fujiwara D, Schreiner EP, Wolff B, Yoshida M, Horinouchi S (1999). Leptomycin B inactivates CRM1/exportin 1 by covalent modification at a cysteine residue in the central conserved region. Proc Natl Acad Sci USA.

[75] Parikh K, Cang S, Sekhri A, Liu D (2014). Selective inhibitors of nuclear export (SINE)—a novel class of anti-cancer agents. J Hematol Oncol..

[76] Neggers JE, Vercruysse T, Jacquemyn M, Vanstreels E, Baloglu E, Shacham S, Crochiere M, Landesman Y, Daelemans D (2015). Identifying drug-target selectivity of small-molecule CRM1/XPO1 inhibitors by CRISPR/Cas9 genome editing. Chem Biol..

[77] Falini B, Mecucci C, Tiacci E, Alcalay M, Rosati R, Pasqualucci L, La Starza R, Diverio D, Colombo E, Santucci A, Bigerna B, Pacini R, Pucciarini A, Liso A, Vignetti M, Fazi P, Meani N, Pettirossi V, Saglio G, Mandelli F, Lo-Coco F, Pelicci PG, Martelli MF, Acute Leukemia Working Party GIMEMA (2005). Cytoplasmic nucleophosmin in acute myelogenous leukemia with a normal karyotype. N Engl J Med.

[78] Falini B, Bolli N, Shan J, Martelli MP, Liso A, Pucciarini A, Bigerna B, Pasqualucci L, Mannucci R, Rosati R, Gorello P, Diverio D, Roti G, Tiacci E, Cazzaniga G, Biondi A, Schnittger S, Haferlach T, Hiddemann W, Martelli MF, Gu W, Mecucci C, Nicoletti I (2006). Both carboxy-terminus NES motif and mutated tryptophan(s) are crucial for aberrant nuclear export of nucleophosmin leukemic mutants in NPMc AML. Blood..

[79] Ranganathan P, Yu X, Na C, Santhanam R, Shacham S, Kauffman M, Walker A, Klisovic R, Blum W, Caligiuri M, Croce CM, Marcucci G, Garzon R (2012). Preclinical activity of a novel CRM1 inhibitor in acute myeloid leukemia. Blood..

[80] Ranganathan P, Kashyap T, Yu X, Meng X, Lai TH, McNeil B, Bhatnagar B, Shacham S, Kauffman M, Dorrance AM, Blum W, Sampath D, Landesman Y, Garzon R (2016). XPO1 inhibition using selinexor synergizes with chemotherapy in acute myeloid leukemia (AML) by targeting DNA repair and restoring topoisomerase II. Clin Cancer Res..

[81] Blum W, Garzon R, Klisovic RB, Schwind S, Walker A, Geyer S, Liu S, Havelange V, Becker H, Schaaf L, Mickle J, Devine H, Kefauver C, Devine SM, Chan KK, Heerema NA, Bloomfield CD, Grever MR, Byrd JC, Villalona-Calero M, Croce CM, Marcucci G (2010). Clinical response and miR-29b predictive significance in older AML patients treated with a 10-day schedule of decitabine. Proc Natl Acad Sci USA.

[82] Ranganathan P, Yu X, Santhanam R, Hofstetter J, Walker A, Walsh K, Bhatnagar B, Klisovic R, Vasu S, Phelps MA, Devine S, Shacham S, Kauffman M, Marcucci G, Blum W, Garzon R (2015). Decitabine priming enhances the antileukemic effects of exportin 1 (XPO1) selective inhibitor selinexor in acute myeloid leukemia. Blood..

[83] Hing ZA, Mantel R, Beckwith KA, Guinn D, Williams E, Smith LL, Williams K, Johnson AJ, Lehman AM, Byrd JC, Woyach JA, Lapalombella R (2015). Selinexor is effective in acquired resistance to ibrutinib and synergizes with ibrutinib in chronic lymphocytic leukemia. Blood..

[84] Turner JG, Kashyap T, Dawson JL, Gomez J, Bauer AA, Grant S, Dai Y, Shain KH, Meads M, Landesman Y, Sullivan DM (2016). XPO1 inhibitor combination therapy with bortezomib or carfilzomib induces nuclear localization of IκBα and overcomes acquired proteasome inhibitor resistance in human multiple myeloma. Oncotarget..

[85] Hing ZA, Fung HYJ, Ranganathan P, Mitchell S, El-Gamal D, Woyach JA, Williams K, Goettl VM, Smith J, Yu X, Meng X, Sun Q, Cagatay T, Lehman AM, Lucas DM, Baloglu E, Shacham S, Kauffman MG, Byrd JC, Chook YM, Garzon R, Lapalombella R (2016). Next-generation XPO1 inhibitor shows improved efficacy and in vivo tolerability in hematological malignancies. Leukemia..

[86] Lapidot T, Sirard C, Vormoor J, Murdoch B, Hoang T, Caceres-Cortes J, Minden M, Paterson B, Caligiuri MA, Dick JE (1994). A cell initiating human acute myeloid leukaemia after transplantation into SCID mice. Nature..

[87] Bonnet D, Dick JE (1997). Human acute myeloid leukemia is organized as a hierarchy that originates from a primitive hematopoietic cell. Nature Med..

[88] Etchin J, Montero J, Berezovskaya A, Le BT, Kentsis A, Christie AL, Conway AS, Chen WC, Reed C, Mansour MR, Ng CE, Adamia S, Rodig SJ, Galinsky IA, Stone RM, Klebanov B, Landesman Y, Kauffman M, Shacham S, Kung AL, Wang JC, Letai A, Look AT (2016). Activity of a selective inhibitor of nuclear export, selinexor(KPT-330), against AML-initiating cells engrafted intoimmunosuppressed NSG mice. Leukemia..

[89] Chen Y, Camacho C, Silvers TR, Razak ARA, Gabrail NY, Gerecitano JF, Kalir E, Pereira E, Evans BR, Ramus SJ, Huang F, Priedigkeit N, Rodriguez E, Donovan M, Khan F, Kalir T, Sebra R, Uzilov A, Chen R, Sinha R, Halpert R, Billaud JN, Shacham S, McCauley D, Landesman Y, Rashal T, Kauffman M, Mirza MR, Mau-Sørensen M, Dottino P, Martignetti JA (2016). Inhibition of the nuclear export receptor XPO1 as a therapeutic target for platinum resistant ovarian cancer. Clin Cancer Res.

[90] Kazim S, Malafa MP, Coppola D, Husain K, Zibadi S, Kashyap T, Crochiere M, Landesman Y, Rashal T, Sullivan DM, Mahipal A (2015). Selective nuclear export inhibitor KPT-330 enhances the antitumor activity of gemcitabine in human pancreatic cancer. Mol Cancer Ther..

[91] Ferreiro-Neira I, Torres NE, Liesenfeld LF, Chan CHF, Penson T, Landesman Y, Senapedis W, Shacham S, Hong TS, Cusack JC (2016). XPO1 inhibition enhances radiation response in preclinical models of rectal cancer. Clin Cancer Res..

[92] Farren MR, Shakya R, Hennessey R, Mace T, Yang J, Elnaggar O, Young G, Landesman Y, Carlson R, Elloul S, Crochiere M, Burd C, Lesinski G (2015). Selinexor, a selective inhibitor of nuclear export (SINE), shows enhanced activity in combination with PD-1/PD-L1 blockade in syngeneic murine models of colon cancer and melanoma. J Immunother Cancer.

[93] Gutierrez M, Goy A, Byrd JC, Flynn JM, Sorensen M, Brown P, Gabrail NY, Savona M, Flinn I, Baz RC, Shah BD, Stone RM, Jacobsen E, Kukreti V, Tiedemann RE, Rashal T, Mirza MR, Shacham S, Kauffman M, Kuruvilla J (2014). A phase 1 dose-escalation study of the oral selective inhibitor of nuclear export (SINE) KPT-330 (selinexor) in patients (pts) with heavily pretreated non-Hodgkin lymphoma (NHL). J Clin Oncol.

[94] Gouder MM, Zer A, Tap WD, Salah S, Dickson MA, Gupta AA, Keohan ML, Loong HH, D’Angelo SP, Baker S, Condy M, Nyquist-Schultz K, Tanner L, Erinjeri JP, Jasmine FH, Friedlander S, Carlson R, Unger TJ, Saint-Martin JR, Rashal T, Ellis J, Kauffman M, Shacham S, Schwartz GK, Abdul Razak AR (2016). Phase IB study of selinexor, a first-in-class inhibitor of nuclear export, in patients with advanced refractory bone or soft tissue sarcoma. J Clin Oncol..

[95] Alexander TB, Lacayo NJ, Choi JK, Ribeiro RC, Pui CH, Rubnitz JE (2016). Phase I study of selinexor, a selective inhibitor of nuclear export, in combination with fludarabine and cytarabine, in pediatric relapsed of refractory acute leukemia. J Clin Oncol..

[96] Monecke T, Haselbach D, Voss B, Russek A, Neumann P, Thomson E, Hurt E, Zachariae U, Stark H, Grubmüller H, Dickmanns A, Ficner R (2013). Structural basis for cooperativity of CRM1 export complex formation. Proc Natl Acad Sci USA.

[97] Port SA, Monecke T, Dickmanns A, Spillner C, Hofele R, Urlaub H, Ficner R, Kehlenbach RH (2015). Structural and functional characterization of CRM1-Nup214 interactions reveals multiple FG-binding sites involved in nuclear export. Cell Rep..

[98] Sun Q, Carrasco YP, Hu Y, Guo X, Mirzaei H, MacMillan J, Chook YM (2013). Nuclear export inhibition through covalent conjugation and hydrolysis of leptomycin B by CRM1. Proc Natl Acad Sci USA.

[99] PubChem compound. https://pubchem.ncbi.nlm.nih.gov/.

[100] Etchin J, Sanda T, Mansour MR, Kentsis A, Montero J, Le BT, Christie AL, McCauley D, Rodig SJ, Kauffman M, Shacham S, Stone R, Letai A, Kung AL, Look TA (2013). KPT-330 inhibitor of CRM1 (XPO1)-mediated nuclear export has selective anti-leukaemic activity in preclinical models of T-cell acute lymphoblastic leukaemia and acute myeloid leukaemia. Br J Haematol..

[101] Azmi AS, Al-Katib A, Aboukameel A, McCauley D, Kauffman M, Shacham S, Mohammad RM (2013). Selective inhibitors of nuclear export for the treatment of non-Hodgkin’s lymphomas. Haematologica..

[102] Walker BA, Wardell CP, Melchor L, Brioli A, Johnson DC, Kaiser MF, Mirabella F, Lopez-Corral L, Humphray S, Murray L, Ross M, Bentley D, Gutiérrez NC, Garcia-Sanz R, San Miguel J, Davies FE, Gonzalez D, Morgan GJ (2014). Intraclonal heterogeneity is a critical early event in the development of myeloma and precedes the development of clinical symptoms. Leukemia..

[103] Zhong Y, El-Gamal D, Dubovsky JA, Beckwith KA, Harrington BK, Williams KE, Goettl VM, Jha S, Mo X, Jones JA, Flynn JM, Maddocks KJ, Andritsos LA, McCauley D, Shacham S, Kauffman M, Byrd JC, Lapalombella R (2014). Selinexor suppresses downstream effectors of B-cell activation, proliferation and migration in chronic lymphocytic leukemia cells. Leukemia..

[104] ClinicalTrials.gov. https://clinicaltrials.gov/.

